# Comprehensive and Efficient Assessment of Psychological Flexibility in the Context of Chronic Pain

**DOI:** 10.1002/ejp.4781

**Published:** 2025-01-06

**Authors:** Amani Lavefjord, Felicia T. A. Sundström, Dane Chia, Fara Tabrizi, Monica Buhrman, Lance M. McCracken

**Affiliations:** ^1^ Department of Psychology Uppsala University Uppsala Sweden; ^2^ Department of Psychology Mid Sweden University Östersund Sweden

**Keywords:** chronic pain, measurement, psychological flexibility, validation

## Abstract

**Background:**

The Multidimensional Psychological Flexibility Inventory (MPFI) is a measure of all facets of psychological flexibility and inflexibility, potentially important processes of change in psychological treatment for chronic pain. In some contexts, it can be considered too long. The aim of this study was, therefore, to validate a short form MPFI (MPFI‐24P) in a chronic pain sample.

**Methods:**

Adults with chronic pain were recruited online (*N* = 404) for a cross‐sectional survey study. They first completed pain background questions and the MPFI. For examining convergent construct validity and explained variance in pain‐related outcomes, participants also completed the Brief Pain Inventory (BPI) Pain Interference Scale, the Work and Social Adjustment Scale (WSAS) and the Patient Health Questionnaire (PHQ‐9), a depression measure. Data were collected on two occasions, 2 weeks apart. Item response theory (IRT) and confirmatory factor analysis (CFA) were used for selecting the best‐performing items.

**Results:**

IRT parameters were overall adequate, and hierarchical CFA demonstrated a good model fit. Network analysis of the MPFI items indicated that, in general, items intended to measure the same facets were substantially interconnected, more so for the inflexibility items. Temporal stability was adequate, and internal consistency was good. The MPFI‐24P correlated with pain interference, work and social adjustment and depression, with the inflexibility scale better predicting these outcomes. The MPFI‐24P correlated strongly with the full‐length MPFI.

**Conclusions:**

The MPFI‐24P for chronic pain is generally valid and reliable, especially the inflexibility scale. It performs similarly to the full‐length MPFI.

**Significance:**

This paper contributes with a measure that is both feasible to use in clinical practice and research, while being able to measure all facets of psychological flexibility and inflexibility—psychological processes of change that are important to evaluate in psychological treatment of chronic pain in order to better individualize treatment.

Psychological treatment for chronic pain, meaning recurrent or persistent pain for at least 3 months, is effective for helping people improve everyday functioning and well‐being (Buhrman, Gordh, and Andersson 2016; Feliu‐Soler et al. 2018; Gandy et al. 2022; Lai et al. 2023). However, effects from standard treatments appear small (Williams et al. 2020), and not everyone will benefit from the same treatment, partly because generalisability between the group and individual level is inexact (Fisher, Medaglia, and Jeronimus 2018; Molenaar and Campbell 2009). People with chronic pain are vastly heterogeneous, and the correlations between variables on an individual level are not automatically the same as those found in group‐level results (Sundström et al. 2024). Hence, a personalised approach to psychological treatment for chronic pain is recommended, based on individual assessment and targeting of relevant psychological processes in treatment for a particular person and their particular goals (McCracken 2023).

Facets of psychological flexibility (PF) represent a set of potentially relevant treatment processes (Hayes et al. 2022; McCracken 2024; Murillo et al. 2022). These facets enable people to engage in daily life in a fulfilling manner in accordance with values and goals, including occasions where thoughts or feelings could exert constraints (Hayes et al. 2013). The facets of PF include acceptance, defusion, self‐as‐context, present moment awareness, values and committed action, and the facets of psychological inflexibility (PI) include experiential avoidance, fusion, self‐as‐content, lack of present moment awareness, lack of connection to values and inaction (Hayes et al. 2013).

Recently, the Multidimensional Psychological Flexibility Inventory (MPFI; Rolffs, Rogge, and Wilson 2018) was created, assessing all facets of PF and PI, enabling identification of which processes merit intervention for a particular person. Since its creation, the MPFI has been validated in several languages (Azadfar et al. 2022; Landi et al. 2021; Lin, Rogge, and Swanson 2020; Pereira et al. 2023; Simkin et al. 2023; Tabrizi et al. 2023) and is valid for use with people with chronic pain (Sundström et al. 2023). However, it contains 60 items, which is likely too burdensome for some. A short form version has been suggested by the original creators (Rolffs, Rogge, and Wilson 2018) and validated (Grégoire et al. 2020), but it is unclear whether this short form is replicable or appropriate specifically for a chronic pain population, something that should be demonstrated so that it can and will be used in this population.

The aim of this study is to derive and validate a short version form of the MPFI (MPFI‐24P) suitable for people with chronic pain. Items will be derived using item response theory (IRT) parameters in combination with factor analysis. Network analysis will be used as a complement, exploring how items relate to each other without the assumption of capturing latent variables. Reliability will be assessed, as well as construct validity in relation to the full‐length MPFI and the outcomes of pain interference, work and social adjustment and depression. The ability of the MPFI‐24P to predict the aforementioned outcomes will be compared to the ability of the full‐length MPFI.

## 1 Methods

### 1.1 Procedure

People with chronic pain were recruited to a cross‐sectional survey study through patient organisations and social media during a time period of 2 months at the end of 2021. This study is based on secondary analyses of data from a previous study, and further details can be found in the earlier report (Sundström et al. 2023). While all pain conditions were included in recruitment, the sample includes particular subsamples of people with fibromyalgia, low back pain and endometriosis, due to recruitment aiming to include conditions that are both commonly studied, such as in the case of low back pain and fibromyalgia, and less commonly studied, as in the case of endometriosis. The main point of this was to allow examination of the generality of results on the group level. Eligibility criteria included fluency in Swedish and access to the internet. After giving their informed consent, participants were asked to fill out multiple questionnaires, and those who did so also received an invitation to participate again 2 weeks later to establish temporal stability. REDCap, a web‐based survey software platform (Harris et al. 2009), was employed. A 2‐week interval was deemed as long enough to prevent participants from simply recalling their exact previous responses but short enough to minimise the risk of greatly altered life circumstances. The within‐subject data collection was enabled by participants consenting to provide their e‐mail addresses. As this study was part of a larger project, participants also responded to more measures than those presented here, for other research purposes not included in this paper. The study was approved by the Swedish Ethical Review Authority (DNR: 2021‐02656).

### 1.2 Measures

#### 1.2.1 Demography and Pain Background

Participants provided their data on age, gender, family origin, work status, education level, relationship status and financial situation. Participants were also asked to report whether they perceived themselves as belonging to a minority group, and if so, whether this caused them any negative consequences in their daily lives. They also reported their pain intensity, currently and during the past week, the duration of their pain, their pain conditions, how frequently they had sought health care due to their pain, which pain treatments they had received, the number of days with pain in a month, their pain sites and whether their pain condition had been medically diagnosed.

#### 1.2.2 Multidimensional Psychological Flexibility Inventory

The MPFI consists of 60 items, with 30 allocated to each of two overarching scales, global flexibility and global inflexibility (Rolffs, Rogge, and Wilson 2018). The global flexibility scale includes the six facets of PF, with five items in each facet. Similarly, the global inflexibility scale includes the six facets of PI, also five items each. All items are rated on a numerical rating scale from one to six (never to always true). A higher mean score indicates greater PF or PI (Rolffs, Rogge, and Wilson 2018).

#### 1.2.3 Items Selected From the Brief Pain Inventory

Nine items were selected from the Brief Pain Inventory (BPI; Cleeland 2009). Two of these measured pain intensity, with one focusing on current pain intensity and one focusing on average pain intensity in the past week. Additionally, seven items assessing pain interference in general activity, mood, walking ability, work, relationships, sleep and enjoyment of life were selected. Again, the time period for responding was the past week. All items are scored on a numerical rating scale from 0 to 10. A higher mean score from these seven items denotes greater pain‐related interference in daily functioning (Cleeland 2009).

#### 1.2.4 Work and Social Adjustment Scale

The Work and Social Adjustment Scale (WSAS; Mundt et al. 2002) consists of five items examining the level of interference from one's condition on functioning at work, home management, leisure activities and relationships. A numerical rating scale from zero to eight is utilised for each item, with a higher total score indicating greater impairment in work and social functioning (Mundt et al. 2002).

#### 1.2.5 Patient Health Questionnaire

The Patient Health Questionnaire (PHQ‐9; Kroenke, Spitzer, and Williams 2001) comprises nine items assessing depression symptoms over the past 2 weeks. A numerical rating scale from zero to three is employed for all items, with a higher total score representing greater severity of depression (Kroenke, Spitzer, and Williams 2001).

### 1.3 Statistical Analyses

Statistical analyses were conducted in JASP 0.16.4 (JASP Team 2022) and R 4.3.0 (R Core Team 2021), using the packages ‘readr’ (Wickham, Hester, and Bryan 2024), ‘mirt’ (Chalmers 2012), ‘mvnormalTest’ (Zhang, Zhou, and Shao 2020), ‘lavaan’ (Rosseel 2012) and ‘semTools’ (Jorgensen et al. 2022).

#### 1.3.1 Data Cleaning

Normality was first examined by looking at histograms and values for skew and kurtosis for all background variables, all items on the MPFI and all summary scores on outcomes. Two background variables—frequency of visiting health care and days in the month with pain—showed little variation and were excluded from further analyses. Multivariate non‐normality was assessed with Mardia's test (Mardia 1970) and accounted for in confirmatory factor analysis (CFA) and network analysis.

Two outliers were identified based on a definition of scores falling above or below three standard deviations (SD) from the mean. These were assessed and deemed as true scores, and thus retained in further analyses in order to not exclude data representing natural variations across people. Due to missing data, pain duration was excluded from further analyses. Other variables, with some missing data, were retained while the data points missing were excluded from analyses. Due to attrition, the number of participants differs across the analyses conducted.

#### 1.3.2 Selecting the MPFI‐24P Items Using Item Response Theory and CFA

IRT analyses were used in combination with subsequent CFA for selecting the items for the MPFI‐24P. IRT refers to a set of analyses used to assess how each individual item in a measure performs (Baker 2001). For example, in relation to the latent variable measured, IRT analyses can indicate whether there are particular items measuring higher or lower levels of this trait, how the response options are utilised, and how well items discriminate between people with high levels of the latent variable and people with lower levels (Nguyen et al. 2014).

Two IRT approaches were employed for selecting items in this study, both employing graded response models (GRM), suitable for measures with several response categories (Nguyen et al. 2014). In the first approach, 12 IRT models were created, one for each facet, in order to evaluate which two items best measured each facet. In the second approach, only two models were created, one for the global flexibility scale and one for the global inflexibility scale, in order to select the two items corresponding to each facet that best measured flexibility and inflexibility. Both of these approaches fulfil assumptions of unidimensionality, but on different levels. In the first approach, each facet is the latent variable in each model. In the second approach, global flexibility and global inflexibility are the overarching latent variables in each model. In both approaches, items were selected on the basis of their so‐called discrimination parameter, where a higher value on this parameter indicates an item that is better able to differentiate between people having higher levels of the variable in question and people having lower levels of the variable, in comparison to other items included in the particular model. While not universally accepted, Baker (2001) proposes guidelines for interpreting the discrimination parameter, where a parameter between 0.01 and 0.34 is considered to be very low, 0.35 to 0.64 is considered to be low, 0.65–1.34 is considered as moderate, 1.35–1.69 is considered high and above 1.7 is considered to be very high.

The items selected from each IRT approach were included in CFA to evaluate the model fit when including data from each of the two approaches. The factor models examined were second‐order models with the items loading on each of the 12 facets of PF and PI, with six of these facets loading on flexibility and six loading on inflexibility. This follows the theoretical framework of PF (Hayes et al. 2013) as well as the empirical evidence in relation to the validation of the full‐length MPFI (Rolffs, Rogge, and Wilson 2018). A Satorra‐Bentler correction for maximum‐likelihood estimation with robust standard errors was used due to multivariate non‐normality.

Goodness of fit was examined by looking at whether the standardised root mean squared residual (SRMR) was below 0.09, whether the root mean square error of approximation (RMSEA) was below 0.08, and whether the comparative fit index (CFI) and the Tucker–Lewis index (TLI) were above 0.90 (Marsh, Hau, and Grayson 2005). Item loadings were deemed as good if they were above 0.32 (Tabachnick and Fidell 2012).

Including data for the items selected in the first approach (based on 12 facets) led to the inclusion of negative variance and Heywood cases in the model, while the inclusion of data for the items selected in the second approach (based on two overarching flexibility and inflexibility dimensions) did not lead to these same results and generated a better model fit. For reference, the originally proposed items for making up a short form version (Rolffs, Rogge, and Wilson 2018) were also evaluated in a CFA in this study, and results once again included negative variance and Heywood cases. Based on this, the selection of items for the MPFI‐24P was based on the second selection approach, prioritising the overarching PF and PI dimensions.

#### 1.3.3 Network Analysis

Network analysis was conducted using graphical least absolute shrinkage and selection operator regularisation, using extended Bayesian information criterion model selection (EBICglasso; Foygel and Drton 2010). The data were first normalised using nonparametric transformation (Liu et al. 2012), due to the data not being multivariate normal.

#### 1.3.4 Reliability

Reliability in terms of temporal stability was evaluated with intraclass correlation coefficients (ICC) between the results from the two survey points in time. Here, we looked at absolute agreement for specific values in a two‐way random‐effect model, with results above 0.5 being moderate and results between 0.75 and 0.9 being good (Koo and Li 2016).

Cronbach's alpha was calculated for examining internal consistency, where an alpha of 0.7–0.95 is deemed as good (Bland and Altman 1997). As an alternative, due to the limitations of Cronbach's alpha (Hayes and Coutts 2020; McNeish 2018; Sijtsma 2009; Trizano‐Hermosilla and Alvarado 2016), the composite reliability statistic, derived from the factor model, is also presented (Green and Yang 2009; Jorgensen et al. 2022).

#### 1.3.5 Background Variables

Pearson correlations, as well as point‐biserial correlations in the case of categorical data, dichotomised where needed, were employed to examine relationships between the background variables and the MPFI‐24P and between the background variables and the pain‐related outcomes of pain interference, work and social adjustment and depression. In order to examine potential differences in the MPFI‐24P based on pain types, a one‐way ANOVA was employed.

#### 1.3.6 Convergent Construct Validity

The results on the MPFI‐24P were assessed by Pearson correlations with the full‐length MPFI, pain interference, work and social adjustment and depression. Correlations above 0.1 were deemed small; correlations above 0.3 were deemed as moderate; and correlations above 0.5 were deemed as strong (Cohen 1988). These correlations were calculated for the whole sample as well as separately for the larger subsamples with people with low back pain, fibromyalgia and endometriosis. The correlations for each subgroup were also subsequently compared to each other using *z*‐scores.

#### 1.3.7 Explained Variance in Pain‐Related Outcomes Compared to the Full‐Length MPFI

Three hierarchical multiple regression (HMR) analyses were employed, one for each of the outcomes: pain interference; work and social adjustment; and depression. First, the background variables of age, having generalised pain, having been prescribed pain medications, education level and pain intensity were entered. In two subsequent steps, the MPFI‐24P flexibility scores were entered, followed by the MPFI‐24P inflexibility scores. These two steps were then replicated but using the full‐length flexibility and full‐length inflexibility scores instead, in order to facilitate comparison of added explained variance of the MPFI‐24P form compared to the full‐length MPFI.

As the background variable pain intensity is measured currently as well as during the past week, only one of these variables was included in analyses. While pain intensity during the past week correlated more strongly with the outcomes, the inclusion of that variable led to a violation of the assumptions of independence of residuals in one step of the analysis, and this variable was instead replaced with current pain intensity. After this adjustment, assumptions for multiple regression were met; multicollinearity and influential outliers were ruled out, residual values were independent, there were linear relationships between predictors and outcomes, and assumptions of normally distributed residuals and homoscedasticity were met. Note that when reporting on the total explained variance of the variables in each model, the adjusted *R* ^2^ was used.

## 2 Results

### 2.1 Participant Characteristics

In total, 404 participants took part in the first timepoint assessment, and 301 participants (74.5%) took part in the study at the second timepoint. The sample was made up mostly of women (93.8%) with a mean age of 47.75 years. Most had family origins from Sweden (83.2%), and most were in a relationship (69.1%). Around half of the participants had some sort of employment (52.3%), a college or university education (55.2%) and a good or very good financial situation (44.8%). People most frequently had pain in their lower back or spine (68.8%), their pelvic region (50.2%), or their neck region (45.5%). Roughly half of the sample had pain generalised beyond a particular body site (54.2%). Although the sample included both conditions typically presenting with chronic pain and endometriosis that may present with more cyclic pain, it can be noted that days of pain per month was very similar across pain conditions. See Table 1 for detailed participant characteristics.

**TABLE 1:** Sample characteristics.

Variables	Percentage or mean and SD
Gender	
Women	93.8
Men	4.5
Transgender men	0.2
Genderqueer/non‐binary	1.2
Does not indicate	0.2
Family origin	
Sweden	83.2
Sweden and other country	4.7
Other countries than Sweden	8.2
Does not indicate	4.0
Minority group identification	
Yes	6.9
No	85.9
Don't know	6.4
Does not indicate	0.7
If yes, this leads to negative effects	63.0
Work status	
Employed full time	34.7
Employed part time	12.9
Self‐employed	4.7
Job seeking	5.4
Sick leave	19.3
Student	5.7
Retired or other	16.5
Does not indicate	0.7
Relationship status	
Single	20.3
Married/in relationship	69.1
Divorced/separated	8.2
Widowed	1.5
Does not indicate	1.0
Financial situation	
Very good	10.1
Good	34.7
Sufficient	35.4
Bad	13.9
Very bad	5.0
Does not indicate	1.0
Highest completed education	
Elementary	5.9
Secondary	32.2
College/university	55.2
Other, such as vocational training	5.9
Does not indicate	0.7
Most frequent pain sites	
Lower back/spine	68.8
Pelvic region	50.2
Neck region	45.5
Medications	
Any medications prescribed	78.0
Prescribed opioids	34.2
Prescribed psychopharmaceutical drugs	43.8
Other	
Have gone through psychological treatment	60.9
Generalised pain	54.2
Pain condition confirmed by medical doctor	91.1
Age in whole sample	M = 47.75, SD = 13.02
Age in low back pain subsample	M = 53.29, SD = 12.08
Age in endometriosis subsample	M = 36.73, SD = 9.70
Age in fibromyalgia subsample	M = 50.53, SD = 11.00
Age in ‘other’ subsample	M = 48.58, SD = 13.23
Health care visits due to pain last six months	M = 4.73, SD = 9.19
Days with pain per month in whole sample	M = 28.14, SD = 4.05
Days with pain per month in low back pain sample	M = 28.42, SD = 3.62
Days with pain per month in endometriosis sample	M = 26.20, SD = 5.32
Days with pain per month in fibromyalgia sample	M = 28.63, SD = 3.42
Days with pain per month in ‘other’ sample	M = 28.76, SD = 3.54

### 2.2 Item Response Theory

Table 2 shows the item discrimination parameters of all selected items. While items in the experiential avoidance facet were on the lower side, remaining parameters appeared adequate. Table 2 also indicates the threshold parameters of each item. In general, a mean level score in PF, or PI, corresponds to choosing one of the response categories towards the middle of the rating scale. For a few items, response patterns were somewhat skewed, in that people who in reality displayed mean level scores chose a response category indicating a higher level of the latent variable in question. Specifically, items measuring self‐as‐context, values and committed action in the flexibility scale did not measure the higher ends of PF as well as the other facet items did.

**TABLE 2:** Selected MPFI‐24P items, their IRT parameters, CFA loadings and intraclass correlations.

Selected items	a	b1	b2	b3	b4	b5	Factor loading (SE)	Intraclass correlation (95% CI)
Psychological flexibility								
*Acceptance*							0.85 (0.41)	
1(2). I tried to make peace with my negative thoughts and feelings rather than resisting them	1.46	−2.91	−1.49	−0.12	1.10	2.52	0.64 (0.09)	0.56 (0.48–0.63)
2(5). I opened myself to all of my feelings, the good and the bad	1.10	−3.01	−1.28	0.24	1.66	3.24	0.55 (0.09)	0.52 (0.43–0.60)
*Present moment awareness*							0.67 (0.14)	
3(7). I was in tune with my thoughts and feelings from moment to moment	1.49	−2.59	−0.73	0.88	1.88	3.03	0.85 (0.07)	0.54 (0.46–0.62)
4(9). I was in touch with the ebb and flow of my thoughts and feelings	1.16	−3.55	−1.39	0.19	1.61	3.03	0.69 (0.06)	0.49 (0.40–0.57)
*Self‐as‐context*							0.85 (0.19)	
5(13). I tried to keep perspective even when life knocked me down	2.55	−2.84	−1.63	−0.57	0.42	1.38	0.88 (0.05)	0.59 (0.51–0.66)
6(14). When I was scared or afraid, I still tried to see the larger picture	2.56	−2.40	−1.23	−0.27	0.76	1.72	0.87 (0.05)	0.60 (0.53–0.67)
*Defusion*							0.83 (0.19)	
7(18). When I was scared or afraid, I was able to gently experience those feelings, allowing them to pass	2.25	−2.11	−0.60	0.54	1.44	2.33	0.84 (0.05)	0.61 (0.54–0.68)
8(20). In tough situations, I was able to notice my thoughts and feelings without getting overwhelmed by them	2.35	−1.86	−0.81	0.28	1.27	2.31	0.84 (0.05)	0.66 (0.59–0.72)
*Values*							0.94 (0.68)	
9(23). I tried to connect with what is truly important to me on a daily basis	2.22	−2.71	−1.59	−0.61	0.34	1.39	0.76 (0.07)	0.59 (0.51–0.66)
10(25). My deeper values consistently gave direction to my life	2.85	−2.39	−1.60	−0.66	0.16	1.09	0.86 (0.08)	0.66 (0.59–0.72)
*Committed action*							0.88 (0.19)	
11(27). Even when times got tough, I was still able to take steps towards what I value in life	2.70	−2.56	−1.53	−0.41	0.49	1.50	0.90 (0.04)	0.60 (0.52–0.67)
12(28). Even when life got stressful and hectic, I still worked towards things that were important to me	2.47	−2.45	−1.46	−0.50	0.64	1.44	0.86 (0.04)	0.59 (0.51–0.66)
Psychological inflexibility								
*Experiential avoidance*							0.30 (0.07)	
13(32). I tried to distract myself when I felt unpleasant emotions	0.58	−7.24	−2.75	0.12	2.49	4.95	0.74 (0.07)	0.61 (0.54–0.68)
14(33). When unpleasant memories came to me, I tried to put them out of my mind	0.71	−4.93	−2.33	−0.08	2.00	3.38	0.95 (0.10)	0.65 (0.57–0.71)
*Lack of contact with the present moment*							0.50 (0.08)	
15(38). I went through most days on auto‐pilot without paying much attention to what I was thinking or feeling	1.07	−2.37	−0.32	0.86	1.98	3.55	0.92 (0.06)	0.62 (0.55–0.69)
16(39). I floated through most days without paying much attention	1.12	−2.24	−0.11	1.20	2.33	4.22	0.93 (0.05)	0.56 (0.48–0.63)
*Self‐as‐content*							0.70 (0.10)	
17(42). I criticised myself for having irrational or inappropriate emotions	2.93	−0.94	0.02	0.91	1.54	2.35	0.93 (0.06)	0.72 (0.66–0.77)
18(43). I believed some of my thoughts are abnormal or bad and I shouldn't think that way	2.86	−1.18	−0.07	0.68	1.45	2.77	0.87 (0.06)	0.67 (0.60–0.72)
*Fusion*							0.85 (0.17)	
19(48). It was very easy to get trapped into unwanted thoughts and feelings	2.89	−1.59	−0.36	0.30	1.10	1.88	0.92 (0.05)	0.69 (0.63–0.75)
20(49). When I had negative thoughts or feelings, it was very hard to see past them	2.79	−1.59	−0.41	0.59	1.24	2.23	0.91 (0.05)	0.67 (0.61–0.73)
*Lack of contact with values*							0.90 (0.35)	
21(51). My priorities and values often fell by the wayside in my day‐to‐day life	2.01	−2.05	−0.34	0.84	1.66	2.56	0.80 (0.06)	0.54 (0.45–0.61)
22(55). When times got tough, it was easy to forget about what I truly value	2.10	−1.55	−0.39	0.67	1.47	2.65	0.80 (0.07)	0.57 (0.48–0.64)
*Inaction*							0.91 (0.30)	
23(57). Negative feelings easily stalled out my plans	2.86	−1.18	−0.07	0.68	1.45	2.77	0.89 (0.05)	0.65 (0.58–0.71)
24(59). Negative experiences derailed me from what's really important	2.93	−0.94	0.02	0.91	1.54	2.35	0.87 (0.05)	0.55 (0.47–0.63)

*Note: N* = 301–404. Item numbers are for the new MPFI‐24P (original MPFI full‐length item number in parenthesis). a = discrimination parameter, indicating how well items differentiate between people with high and low levels of psychological flexibility or inflexibility. b1 = threshold parameter indicating the standardised latent variable level where there is a 50% probability that respondents will select the first response category and 50% probability of selecting the second response category. b2–b5 = additional threshold parameters, indicating the 50% probabilities of selecting the second and third response categories (b2), the third and fourth response categories (b3), the fourth and fifth response categories (b4) and the fifth and sixth response categories (b5). All factor loadings were significant at *p* < 0.001.

### 2.3 Confirmatory Factor Analysis

CFA indicated a good model fit (*χ* ^2^_(df=239)_ = 530.22; *p* < 0.001; robust CFI = 0.94; robust TLI = 0.94; SRMR = 0.06; robust RMSEA = 0.06) for the MPFI‐24P, with mostly adequate factor loadings on both first‐order factors and second‐order factors. One exception was the first‐order factor experiential avoidance that had a somewhat lower loading on the second‐order factor PI. See Table 2 for details.

### 2.4 Network Analysis

**FIGURE 1:**
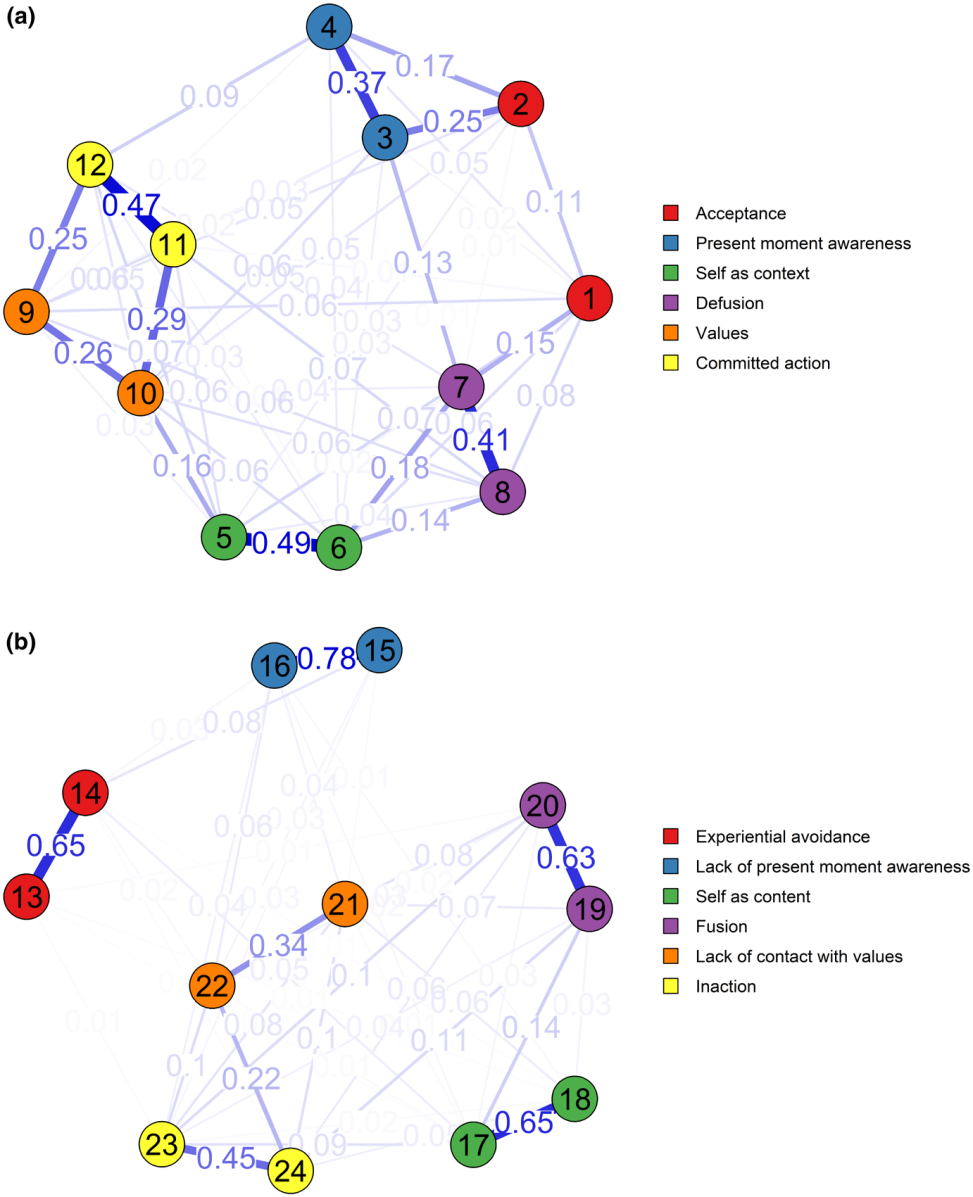
(a, b) Network illustrations of how the flexibility items (a) and the inflexibility items (b) of the MPFI‐24P are interconnected. The circles illustrate the item variables, and the thickness of edges between the circles illustrate the strength of the correlation between variables.

The flexibility items belonging to the same facet were, in general, relatively well associated with each other. However, the values items had a lower correlation with each other and appeared equally connected to the committed action items as they were to each other. Further, the two acceptance items did not appear well connected. In particular, the first acceptance item did not connect well to any of the other items in the scale. See Figure 1. The items showing the highest levels of expected influence centrality were self‐as‐context item 6 (*z* = 1.08), values item 10 (*z* = 1.00) and committed action item 12 (*z* = 0.81).

For the inflexibility items, all item pairs belonging to the same facet were in general well connected, especially the lack of present moment awareness items, the self‐as‐content items, the fusion items and the lack of contact with values items. The remaining connections of items between different facets were weaker. See Figure 1b. The items showing the highest levels of expected influence centrality were self‐as‐content item 17 (*z* = 1.05), fusion item 19 (*z* = 1.11) and inaction item 24 (*z* = 0.86).

### 2.5 Reliability

Intraclass correlations were mostly moderate, although one present‐moment awareness item was just below the cut‐off for moderate. See Table 2. Overall, internal consistency was good, both for items measuring particular facets and for items measuring the global scales of flexibility and inflexibility. One exception was the acceptance facet, where internal consistency was low. See Table 3.

**TABLE 3:** Cronbach's alpha and composite reliability.

Scale	Cronbach's alpha	Composite reliability
*Psychological flexibility*	0.91	0.95
Acceptance	0.52	0.52
Present moment awareness	0.74	0.74
Self‐as‐context	0.86	0.87
Defusion	0.82	0.83
Values	0.79	0.79
Committed action	0.87	0.87
*Psychological inflexibility*	0.89	0.91
Experiential avoidance	0.82	0.85
Lack of contact with the present moment	0.92	0.92
Self‐as‐content	0.89	0.89
Fusion	0.91	0.91
Lack of contact with values	0.78	0.78
Inaction	0.87	0.87

*Note: N* = 404.

### 2.6 Background Variables

Age had a small positive correlation with the MPFI‐24P flexibility scale, small negative correlations with the MPFI‐24P inflexibility scale and with pain interference, and a medium‐sized negative correlation with depression. Identifying as being part of a minority group was, to a small degree, correlated with higher scores of depression as well as higher scores on the MPFI‐24P inflexibility scale.

Having a history of being prescribed mood or anxiety medication and going through psychological treatment was associated with lower scores on the MPFI‐24P flexibility scale, higher scores on the MPFI‐24P inflexibility scale and higher depression scores, to a small degree. A history of going through psychological treatment also had a small positive correlation with pain interference.

Having generalised pain, having been prescribed pain medications in general, and opioids in particular, and having had one's pain diagnosis confirmed by a medical doctor were all associated with more pain interference and lower work and social adjustment, to a small degree. Having generalised pain and having been prescribed opioids was also associated with higher levels of depression, again to a small degree. However, these variables did not correlate with the MPFI‐24P flexibility or inflexibility scales.

Having any kind of employment and having a better financial situation were weakly related to having less pain interference, higher work and social adjustment and less depression. Having a university education was related to all outcomes except for work and social adjustment. None of these background variables correlated with the MPFI‐24P scales. See Table 4 for detailed correlations. Lastly, one‐way analyses of variance showed no difference between pain types on scores on the MPFI‐24P flexibility scale (*F* = 0.75; *p* = 0.97) or the inflexibility scale (F = 0.64, *p* = 0.59). See Table 5 for descriptive statistics per pain type.

**TABLE 4:** Correlation analyses between background variables and the MPFI‐24P flexibility and inflexibility scales and between background variable and the outcomes.

Background variables	Flexibility	Inflexibility	BPI pain interference	WSAS	PHQ‐9
*Continuous variables*					
Pain intensity during last week	−0.05	0.21^c^	0.55^c^	0.42^c^	0.30^c^
Current pain intensity	−0.02	0.25^c^	0.42^c^	0.34^c^	0.30^c^
Age	0.13^b^	−0.21^c^	−0.11^a^	−0.09	−0.30^c^
*Dichotomous pain variables*					
Generalised pain	−0.02	−0.06	−0.14^b^	−0.16^b^	−0.13^a^
Pain medications prescription	0.07	−0.01	−0.20^c^	−0.24^c^	−0.08
Opioids prescription	0.05	−0.05	−0.19^c^	−0.20^c^	−0.12^b^
Mood or anxiety drugs prescription	0.23^c^	−0.25^c^	−0.09	−0.08	−0.19^c^
Psychological treatment	0.17^c^	−0.23^c^	−0.16^b^	−0.09	−0.22^c^
Pain diagnosis confirmed by medical doctor	−0.02	0.05	−0.17^c^	−0.24^c^	−0.04
*Dichotomous demography variables*					
Minority group identification	0.01	−0.10^a^	−0.01	0.02	−0.10^a^
Relationship status	−0.01	−0.07	0.02	−0.08	−0.03
Work status	−0.05	0.07	0.10^a^	0.29^c^	0.12^a^
Financial situation	−0.07	0.08	0.14^b^	0.24^c^	0.16^b^
Education level	−0.02	0.08	0.12^a^	0.09	0.12^a^

*Note: N* = 386–404. Dichotomous pain variables: yes = 1, no/don't know = 2. Dichotomous demography variables: yes = 1, no/don't know = 2 for minority group identification. Single/divorced/widowed = 1, relationship/married = 2 for relationship status. Any employment = 1, sick leave/student/unemployed/retired = 2 for work status. Very good/good = 1, sufficient/bad/very bad = 2 for financial situation. Attended university = 1, did not attend university = 2 for educational level. BPI = Brief Pain Inventory. WSAS = work and social adjustment scale. PHQ‐9 = Patient Health Questionnire‐9.

^a^ *p* < 0.05.

^b^ *p* < 0.01.

^c^ *p* < 0.001.

**TABLE 5:** Sub‐group descriptive statistics for MPFI‐24P scores.

Condition	*N*	Mean flexibility scores (SD)	Mean inflexibility scores (SD)
Low back pain	85	3.63 (0.78)	2.85 (0.74)
Endometriosis	81	3.61 (0.81)	3.04 (0.91)
Fibromyalgia	116	3.64 (0.88)	2.97 (0.88)
Other	122	3.67 (0.85)	2.97 (0.86)
Total	404	3.64 (0.83)	2.96 (0.85)

### 2.7 Convergent Construct Validity

Correlation values between the various MPFI‐24P scales are included in Table 6, along with correlation values between the MPFI‐24P scales and the full‐length MPFI global scales and between the MPFI‐24P scales and the three outcomes measured by BPI, WSAS and PHQ‐9.

**TABLE 6:** Correlations between the global and sub‐scales of the MPFI‐24P, global scales of the full‐length MPFI and outcome variables.

Scale	Flexibility (24P)	Flexibility (full‐length)	Acceptance	Present moment awareness	Self as context	Defusion	Values	Committed action	Inflexibility (24P)	Inflexibility (full‐length)	Experiential avoidance	Lack of contact with the present moment	Self as content	Fusion	Lack of contact with values	Inaction
Flexibility (full‐length)	0.98^b^	—														
Acceptance	0.74^b^	0.73^b^	—													
Present moment awareness	0.70^b^	0.70^b^	0.57^b^	—												
Self‐as‐context	0.83^b^	0.80^b^	0.50^b^	0.45^b^	—											
Defusion	0.80^b^	0.79^b^	0.52^b^	0.45^b^	0.67^b^	—										
Values	0.84^b^	0.82^b^	0.50^b^	0.45^b^	0.64^b^	0.58^b^	—									
Committed action	0.82^b^	0.80^b^	0.43^b^	0.46^b^	0.63^b^	0.56^b^	0.76^b^	—								
Inflexibility (24P)	−0.58^b^	−0.57^b^	−0.38^b^	−0.36^b^	−0.44^b^	−0.49^b^	−0.54^b^	−0.52^b^	—							
Inflexibility (full‐length)	−0.59^b^	−0.58^b^	−0.39^b^	−0.36^b^	−0.44^b^	−0.50^b^	−0.54^b^	−0.52^b^	0.99^b^	—						
Experiential avoidance	−0.08	−0.09	−0.17^b^	−0.10	0.02	−0.05	−0.05	−0.04	0.50^b^	0.51^b^	—					
Lack of contact with the present moment	−0.31^b^	−0.30^b^	−0.19^b^	−0.29^b^	−0.24^b^	−0.20^b^	−0.29^b^	−0.26^b^	0.64^b^	0.62^b^	0.29^b^	—				
Self‐as‐content	−0.43^b^	−0.42^b^	−0.30^b^	−0.32^b^	−0.30^b^	−0.39^b^	−0.38^b^	−0.34^b^	0.77^b^	0.75^b^	0.27^b^	0.34^b^	—			
Fusion	−0.62^b^	−0.61^b^	−0.39^b^	−0.33^b^	−0.51^b^	−0.60^b^	−0.55^b^	−0.53^b^	0.79^b^	0.78^b^	0.20^b^	0.31^b^	0.58^b^	—		
Lack of contact with values	−0.52^b^	−0.51^b^	−0.29^b^	−0.25^b^	−0.43^b^	−0.38^b^	−0.54^b^	−0.55^b^	0.79^b^	0.77^b^	0.22^b^	0.44^b^	0.51^b^	0.60^b^	—	
Inaction	−0.54^b^	−0.54^b^	−0.32^b^	−0.26^b^	−0.43^b^	−0.46^b^	−0.51^b^	−0.55^b^	0.83^b^	0.82^b^	0.24^b^	0.41^b^	0.55^b^	0.69^b^	0.70^b^	—
BPI‐PI	−0.20^b^	−0.22^b^	−0.13^b^	−0.09	−0.13^b^	−0.20^b^	−0.21^b^	−0.19^b^	0.39^b^	0.40^b^	0.21^b^	0.23^b^	0.28^b^	0.31^b^	0.34^b^	0.33^b^
WSAS	−0.15^b^	−0.16^b^	−0.1^a^	0.07	−0.08	−0.16^b^	−0.16^b^	−0.19^b^	0.31^b^	0.31^b^	0.17^b^	0.24^b^	0.18^b^	0.17^b^	0.31^b^	0.30^b^
PHQ‐9	−0.42^b^	−0.41^b^	−0.28^b^	−0.26^b^	−0.29^b^	−0.36^b^	−0.40^b^	−0.37^b^	0.64^b^	0.64^b^	0.19^b^	0.38^b^	0.54^b^	0.56^b^	0.53^b^	0.55^b^

*Note: N* = 395–404.

^a^ *p* < 0.05.

^b^ *p* < 0.01.

#### 2.7.1 Facet‐Level Correlations

The flexibility facet subscales had medium to strong positive correlations with each other. The inflexibility facet subscales also mostly had medium to strong positive correlations with each other, although experiential avoidance in particular had lower positive correlations with the other inflexibility subscales.

The inflexibility facet subscales often had low to medium negative correlations with the flexibility facet subscales. However, some strong negative correlations also occurred, especially between the inflexibility facet fusion and most of the flexibility subscales, and between the flexibility subscales of values and committed action on the one hand, and their inflexibility counterparts, lack of contact with values and inaction on the other hand. Notably, the inflexibility subscale of experiential avoidance mostly lacked significant correlations with the flexibility subscales.

#### 2.7.2 Global Scale‐Level Correlations

A near‐perfect positive correlation was found for the MPFI‐24P global flexibility scale and the full‐length global flexibility scale. This was also the case for the MPFI‐24P global inflexibility scale and the full‐length global inflexibility scale.

The MPFI‐24P global flexibility scale showed strong negative correlations with the global inflexibility scale in both the MPFI‐24P and in the full‐length measure. Likewise, the MPFI‐24P global inflexibility scale had a strong negative correlation with the MPFI‐24P flexibility scale, as already indicated, but also with the full‐length global flexibility scale.

#### 2.7.3 Correlations Between Facets and Global Scales

All flexibility facet level subscales of the new MPFI‐24P had strong positive correlations with the MPFI‐24P global scale of flexibility. Very similar correlation values were found between the flexibility subscales and the global scale from the originally validated full‐length MPFI. Following the same pattern, the MPFI‐24P inflexibility subscales correlated strongly and positively with the global scale of inflexibility in the MPFI‐24P as well as in the full‐length MPFI.

The MPFI‐24P global flexibility scale mostly had medium to strong negative correlations with the facet‐level subscales of inflexibility, although no correlations were seen with experiential avoidance in particular. Once again, the correlation values in principle replicated those found between the full‐length global flexibility scale and the facet‐level subscales of inflexibility. The MPFI‐24P global inflexibility scale had medium to strong negative correlations with the flexibility facet subscales, and again, the correlation values replicated those seen between the full‐length global inflexibility scale and the flexibility facet subscales.

#### 2.7.4 MPFI Correlations With Outcomes

The MPFI‐24P global flexibility scale had a moderate negative correlation with PHQ‐9 and small negative correlations with WSAS and BPI‐PI. The MPFI‐24P global inflexibility scale showed a high positive correlation with PHQ‐9 and medium correlations with WSAS and BPI‐PI. The correlation values mimicked those seen between these outcomes and the full‐length MPFI global scales.

At the flexibility facet level, the flexibility facets of acceptance, present moment awareness and self‐as‐context had small negative correlations with all outcomes. Defusion, values and committed action had a medium negative correlation with PHQ‐9 but low negative correlations with WSAS and BPI‐PI. The inflexibility facet of experiential avoidance had small positive correlations with all outcomes, while lack of contact with the present moment had medium positive correlations with all outcomes. Self‐as‐content, fusion, lack of contact with values and inaction all had strong positive correlations with PHQ‐9 and small‐ to medium‐sized positive correlations with the other outcomes.

### 2.8 Explained Variance in Pain‐Related Outcomes Compared to the Full‐Length MPFI

#### 2.8.1 Pain Interference

Entering all background variables into the model led to an adjusted explained variance of 23.9% in pain interference (*F*_(df=5377)_ = 23.73; *p* < 0.001). Entering the scores from the MPFI‐24P flexibility scale in a second model increased the explained variance by 3.3% (*F*_(df=1376)_ = 17.19; *p* < 0.001), with the model now explaining 26.1% of the variance (*F*_(df=6376)_ = 23.49; *p* < 0.001). Finally, entering the scores from the MPFI‐24P inflexibility scale in a third model, increased the explained variance with another 4.5% (*F*_(df=1375)_ = 24.57; *p* < 0.001), with this last model explaining 30.5% of the total variance (*F*_(df=5375)_ = 24.91; *p* < 0.001). In the last model, only pain intensity, PI and having been prescribed pain medications were significant predictors of pain interference, in that order.

In an alternative second model, where the full‐length MPFI flexibility scale scores were added instead of the MPFI‐24P scores after the background variables, the explained variance increased by 4% (*F*_(df=1376)_ = 20.91; *p* < 0.001), with the second model explaining 26.8% of the variance instead (*F*_(df=6376)_ = 24.31; *p* < 0.001). In an alternative third model, entering the full‐length MPFI inflexibility scale scores, the explained variance increased by 4.5% (*F*_(df=1375)_ = 24.86; *p* < 0.001), with a total explained variance of 31.2% (*F*_(df=7375)_ = 25.71; *p* < 0.001). In this last model, again, the strongest predictor was pain intensity, followed by PI and having been prescribed pain medications. See Table 7 for details.

**TABLE 7:** Hierarchical multiple regression for predicting pain‐related outcomes.

Models	Pain interference			Work and social adjustment			Depression		
	*R* ^2^	∆*R* ^2^	*β*	*R* ^2^	∆*R* ^2^	*β*	*R* ^2^	∆*R* ^2^	*β*
*Model 1*									
Age	0.23^c^		−0.12^b^	0.18^c^		−0.09^a^	0.20^c^		−0.13^c^
Generalised pain			−0.09^a^			−0.12^a^			−0.11^a^
Pain medications			−0.16^c^			−0.23^c^			−0.04
Education level			0.06			0.03			0.06
Pain intensity			0.4^c^			0.31^c^			0.29^c^
*Model 2*									
Age	0.26^c^	0.03^c^	−0.09^a^	0.20^c^	0.02^b^	−0.08	0.34^c^	0.14^c^	−0.25^c^
Generalised pain			−0.09^a^			−0.11^a^			−0.11^a^
Pain medications			−0.15^c^			−0.22^c^			−0.03
Education level			0.05			0.03			0.05
Pain intensity			0.40^c^			0.30^c^			0.29^c^
MPFI 24P flexibility			−0.19^c^			−0.13^b^			−0.38^c^
*Model 3*									
Age	0.31^c^	0.05^c^	−0.05	0.23^c^	0.04^c^	−0.04	0.47^c^	0.13^c^	−0.18^c^
Generalised pain			−0.08			−0.10^a^			−0.09^a^
Pain medications			−0.17^c^			−0.24^c^			−0.07
Education level			0.05			0.02			0.04
Pain intensity			0.33^c^			0.25^c^			0.18^c^
MPFI 24P flexibility			−0.02			0.01			−0.12^a^
MPFI 24P inflexibility			−0.28^c^			0.25^c^			0.47^c^
*Alt. model 2*									
Age	0.27^c^	0.04^c^	−0.10^a^	0.20^c^	0.02^b^	−0.08	0.35^c^	0.15^c^	−0.26^c^
Generalised pain			−0.09^a^			−0.16^a^			−0.11^a^
Pain medications			−0.15^c^			−0.21^c^			−0.01
Education level			0.05			0.03			0.05
Pain intensity			0.40^c^			0.31^c^			0.30^c^
MPFI full‐length flexibility			−0.20^c^			−0.14^b^			−0.29^c^
*Alt. model 3*									
Age	0.31^c^	0.05^c^	−0.05	0.23^c^	0.03^c^	−0.035	0.47^c^	0.12^c^	−0.17^c^
Generalised pain			−0.08			−0.10^a^			−0.09^a^
Pain medications			−0.17^c^			−0.24^c^			−0.07
Education level			0.04			0.02			0.03
Pain intensity			0.34^c^			0.25^c^			0.20^c^
MPFI full‐length flexibility			−0.04			−0.01			−0.12^a^
MPFI full‐length inflexibility			0.28^c^			0.24^c^			0.47^c^

*Note: N* = 376–383. *R* ^2^ = adjusted *R* square; ∆*R* ^2^ = *R* square change; *β* = Standardised regression coefficient.

^a^ *p* < 0.05.

^b^ *p* < 0.01.

^c^ *p* < 0.001.

#### 2.8.2 Work and Social Adjustment

In the first model, containing all background variables, the adjusted explained variance in work and social adjustment was 18.1% (*F*_(df=5372)_ = 17.71; *p* < 0.001). In the second model, entering the scores from the MPFI‐24P flexibility scale increased the explained variance by 5.1% (*F*_(df=2370)_ = 12.41; *p* < 0.001), leading to the model now explaining 22.9% of the variance (*F*_(df=7370)_ = 16.98; *p* < 0.001). Entering the scores from the MPFI‐24P inflexibility scale in the third model increased the explained variance with another 3.5% (*F*_(df=1370)_ = 16.88; *p* < 0.001), with this last model explaining 22.9% of the total variance in work and social adjustment (*F*_(df=7370)_ = 16.98; *p* < 0.001). In the last model, pain intensity and PI were the strongest predictors, followed by having been prescribed pain medications and having generalised pain.

In an alternative second model, including the full‐length MPFI flexibility scale scores instead of the MPFI‐24P scores, the explained variance increased by 1.9% (*F*_(df=1371)_ = 9.02; *p* = 0.003), with the second model explaining 19.9% of the variance instead (*F*_(df=6371)_ = 16.58; *p* < 0.001). Entering the full‐length MPFI inflexibility scale scores in an alternative third model, the explained variance increased by 3.2% (*F*_(df=1370)_ = 15.67; *p* < 0.001), with a total explained variance of 22.9% (*F*_(df=7370)_ = 17.02; *p* < 0.001). In this last model, the strongest predictor was pain intensity, followed by PF and having been prescribed pain medications. See Table 7 for details.

#### 2.8.3 Depression

In the first model with all background variables included, the explained variance in depression was 20.2% (*F*_(df=5370)_ = 19.98; *p* < 0.001). Entering the scores from the MPFI‐24P flexibility scale in a second model increased the explained variance by 14.2% (*F*_(df=1369)_ = 81.25; *p* < 0.001), with the model now explaining 34.4% of the variance when (*F*_(df=6369)_ = 33.80; *p* < 0.001). Entering the scores from the MPFI‐24P inflexibility scale in a third model increased the explained variance with an additional 12.5% (*F*_(df=1368)_ = 88.52; *p* < 0.001), with this last model explaining 47% of the variance (*F*_(df=7368)_ = 48.49; *p* < 0.001). In the last model, PI was the most significant predictor, followed by pain intensity and age, and then PF and lastly generalised pain.

In an alternative second model, where the full‐length MPFI flexibility scale scores were added, the explained variance increased by 14.7% (*F*_(df=1369)_ = 84.83; *p* < 0.001), with the second model explaining 34.9% of the variance (*F*_(df=6369)_ = 34.56; *p* < 0.001). In an alternative third model, entering the full‐length MPFI inflexibility scale scores, the explained variance increased by 12.1% (*F*_(df=1368)_ = 85.35; *p* < 0.001), with a total explained variance of 47% (*F*_(df=7368)_ = 48.58; *p* < 0.001). In the last model, PI was the most significant predictor, followed by pain intensity, age, PF and lastly generalised pain. See Table 7 for details.

## 3 Discussion

The aim of this study was to address the need for a comprehensive and efficient measure of psychological flexibility specifically in the context of chronic pain. Here we derive and validate a short form MPFI suitable for this. The items selected in general showed adequate item discrimination parameters. With few exceptions, participants with mean levels of PF or PI tended to select a response in the middle of the response scale, indicating that the rating scales are, in general, adequate.

CFA showed a good model fit with overall adequate factor loadings, although experiential avoidance had a marginally adequate loading on PI, meaning that this particular inflexibility facet subscale is relatively less successful in reflecting PI. Looking at the network analysis, the most central flexibility items were self‐as‐context item 6, values item 10 and committed action item 12, while the most central inflexibility items were self‐as‐content item 17, fusion item 19 and inaction item 24, indicating that these items are especially important in capturing global flexibility and global inflexibility. This is also reflected in higher IRT discrimination parameters for these items. In general, items belonging to the same facets were more strongly connected to each other than to the other items in the networks, particularly in the PI item network. In the flexibility network, the two acceptance items were not strongly connected to each other, indicating that these items might be measuring two somewhat unrelated phenomena. Further, the values items were as connected to the committed action items as they were to each other.

We observe a discrepancy between the network analysis results and CFA results. In the CFA, the acceptance items and values items all had adequate loadings on their corresponding latent variables, while in the network analyses neither the acceptance nor the values items were strongly interconnected to each other. Whereas the CFA examines a predetermined model specifically testing item or facet loadings on latent variables, the network analysis is more exploratory in nature, not restricted to a specific model structure to be tested, and shows correlation values that control for all other correlations in the network. While the items seem to be measuring similar latent variables in the CFA, the items in question may be more, or similarly, strongly correlated with other items than with their specific facet companion. This is perhaps not surprising given that the selection process was based on finding items best measuring the overall constructs of PF or PI.

The weak correlation between the acceptance items in the network analysis is also reflected in low internal consistency for the acceptance facet. Internal consistency was good for all other facets and for the global flexibility and inflexibility scales. Test–retest reliability of items was generally good.

We note that correlation and regression results for the MPFI‐24P global scales in relation to the outcomes measured by BPI‐PI, WSAS and PHQ‐9 very closely resembled results from the full‐length global scales and these same outcomes. This highlights the practical utility of the shorter measure. Also, the inflexibility scales, whether from the MPFI‐24P or full‐length MPFI, predict the outcomes better than the flexibility scales.

All in all, the MPFI‐24P seems to be generally valid and reliable. Importantly, both the global scales and the facet scales perform in a very similar manner to the full‐length MPFI. As in the full‐length MPFI (Sundström et al. 2023; Tabrizi et al. 2023), the inflexibility scale seems to outperform the flexibility scale. In the flexibility scale, items measuring self‐as‐context, values and committed action did not seem to measure the higher ends of PF. Clinicians should thus keep in mind that a person selecting a middle response option on these items may in fact be presenting with less than average levels of PF. Further, assessing potential improvements over time could be difficult if the scales do not measure the higher ends, and we recommend using the corresponding inflexibility scales if these particular facets need to be evaluated over time.

The low internal consistency in the acceptance facet, also reflected within network analysis, should also be addressed. We do not recommend using the acceptance facet in isolation, but if it is, it may be interesting to discuss with the patient what the items mean for them, particularly in a clinical one‐to‐one basis. This could help to determine how they understand the items, in order to better appreciate their behaviour in relation to this facet and how it relates with the overall global concept of psychological flexibility. In the future, some reformulation of acceptance items may be needed.

Using the inflexibility scale, one needs to keep in mind that the experiential avoidance facet in particular had a lower loading on inflexibility, although close to the cutoff for good loading – a result mimicking what was found in relation to the full‐length measure (Sundström et al. 2023). This facet also showed weaker correlations with other inflexibility facets. The experiential avoidance facet may not be the best facet to use as stand‐alone items if one wishes to select particular facet items to administer. Again, a clinician wishing to administer this facet on its own might supplement the item response data with additional discussion with the client. Remaining facets of the inflexibility scale, and both global scales in full, are deemed as adequate for further use, and as indicated above, the inflexibility scale may perform better.

The current study has several limitations, including a sample mostly consisting of women, missing data in some background variables, and, of course, the fact that the study is a cross‐sectional survey study and thus indicative of correlation rather than causation. There was also attrition between the two time points, due to unknown reasons. Items were selected based on how well they measured the overarching constructs of flexibility or inflexibility, meaning that the items selected for each facet are not necessarily the items that best measured each particular facet. However, there is value in capturing flexibility and inflexibility considered broadly, and the other approach did not yield satisfactory outcomes. In the current study, IRT is mainly used for item selection, along with examining threshold parameters. While this may not utilise the potential of IRT in full, it did fulfil an important purpose that could not as easily be fulfilled by classical test theory analyses.

Strengths of the study include multiple pain conditions and pain conditions often confirmed by a medical doctor. However, a discussion of the inclusion of both conditions characterised by continuous chronic pain and conditions such as endometriosis that are sometimes characterised by cyclical pain is warranted. The risk of including both types of conditions is that this creates heterogeneity, making it unclear to which conditions and persons these results apply. However, as seen in Table 1, the number of days with pain was very similar across pain types. We also compared MPFI‐24P scores between pain types and observed no differences. Still, heterogeneity in this study may somewhat obscure the question of to whom results apply. Then again, individual differences may be more prominent than differences between pain conditions (Sundström et al. 2024).

Our heterogeneous sample also included people of various ages, and older age was related to higher flexibility levels and lower inflexibility levels. While this might suggest that psychological flexibility naturally increases as one grows older, it could also indicate that these concepts present themselves differently in older people. It may be that we need a separate measure for an older population, where items are formulated differently.

To conclude, the MPFI‐24P is generally valid and reliable to use in a chronic pain population, especially the inflexibility scale. At the same time, it is important to further consider some of the item content. Many items appear rather abstract. This is understandable given the abstract concepts being measured, but these items might be difficult for respondents to understand or to apply to themselves. Future research could look into whether some item content needs to be refined and particularly address the shortcomings of the experiential avoidance and acceptance facets. While the MPFI is a substantial step forward in regards to assessing PF and PI, identified limitations in performance suggest that further instrument development in the future is warranted.

While the current study shows that the MPFI‐24P for chronic pain is valid on a general group level, this does not necessarily mean that the measure is going to be valid for every individual. We think this is a consideration that is important as we increasingly realise the limitations of aggregated group data and the need to personalise our focus on the process of change and treatment delivery (Hayes et al. 2022; McCracken 2023). The items forming the MPFI‐24P can be seen as a starting point for future work aiming to generate items that could be adapted for use in repeated measures on an individual level. This approach is highly recommended given the wider purposes connected to the flexibility/inflexibility model, including better treatment results for individual people.

## Author Contributions

A.L., F.T.A.S., M.B. and L.M.M. were involved in conceptualising the study. All authors were involved in discussing the results and drafting or revising the manuscript.

## Conflicts of Interest

The authors declare no conflicts of interest.
